# Clinical and molecular features of four Brazilian families with multiple endocrine neoplasia type 1

**DOI:** 10.3389/fendo.2023.1117873

**Published:** 2023-03-10

**Authors:** Isabella Santiago de Melo Miranda, Luciana Pinto Valadares, Gustavo Barcelos Barra, Pedro Góes Mesquita, Lidiana Bandeira de Santana, Lucas Faria de Castro, Ticiane Henriques Santa Rita, Luciana Ansaneli Naves

**Affiliations:** ^1^ Section of Endocrinology, University Hospital of Brasilia, Brasilia, Brazil; ^2^ SARAH Network Rehabilitation Hospitals, Brasilia, Brazil; ^3^ Genomics Section, Sabin Medicina Diagnóstica, Brasília, Brazil

**Keywords:** multiple endocrine neoplasia type 1, primary hyperparathyroidism, pituitary adenoma, gastroenteropancreatic tumor, menin gene, variants, molecular test

## Abstract

**Objective:**

Multiple endocrine neoplasia type 1 (MEN1) is an autosomal dominant syndrome characterized by its clinical variability and complexity in diagnosis and treatment. We performed both clinical and molecular descriptions of four families with MEN1 in a follow-up at a tertiary center in Brasília.

**Methods:**

From a preliminary review of approximately 500 medical records of patients with pituitary neuroendocrine tumor (PitNET) from the database of the Neuroendocrinology Outpatient Clinic of the University Hospital of Brasília, a total of 135 patients met the criteria of at least two affected family members. From this cohort, we have identified 34 families: only four with a phenotype of MEN1 and the other 30 families with the phenotype of familial isolated pituitary adenoma (FIPA). Eleven patients with a clinical diagnosis of MEN1 from these four families were selected.

**Results:**

Variants in *MEN1* gene were identified in all families. One individual from each family underwent genetic testing using targeted high-throughput sequencing (HTS). All patients had primary hyperparathyroidism (PHPT), and the second most common manifestation was PitNET. One individual had well-differentiated liposarcoma, which has been previously reported in a single case of MEN1. Three variants previously described in the database and a novel variant in exon 2 have been found.

**Conclusions:**

The study allowed the genotypic and phenotypic characterization of families with MEN1 in a follow-up at a tertiary center in Brasília.

## Introduction

Multiple endocrine neoplasia type 1 (MEN1; OMIM 131100) is an autosomal dominant syndrome characterized by its clinical variability and complexity in diagnosis and treatment. It is primarily defined by the occurrence of tumors in the parathyroid glands, gastroenteropancreatic (GEP) tract, and anterior pituitary (1, 2). Other endocrine and non-endocrine tumors can also occur, such as adrenocortical adenomas, carcinoids, angiofibromas, collagenomas, leiomyomas, and lipomas (3, 4). *MEN1* gene contains 10 exons that encode a 610-amino-acid protein called MENIN, which acts as a tumor suppressor. MENIN seems to play a role in regulating DNA replication and transcription and in maintaining the integrity of the genome (5).

The delay in diagnosis occurs in most cases. These patients may already present with complications related to these tumors, increasing morbidity and mortality, thus leading to a worse prognosis (2). Therefore, it is important to recognize this great phenotypic variability.

Although there is no well-established genotype–phenotype correlation, genetic testing should be performed to confirm clinical diagnosis and identify asymptomatic first-degree relatives (2, 6). The absence of hot spots establishes the need for sequencing the entire *MEN1* gene. Genetic evaluation using targeted high-throughput sequencing (HTS) analyses multiple coding regions and splicing sites simultaneously, in addition to having a lower cost when compared to the Sanger method (7, 8).

For this purpose, we performed both clinical and molecular descriptions of four families with MEN1 in a follow-up at a tertiary center in Brasília. The aim of this study is to emphasize the importance of recognizing the several clinical manifestations that may be present in individuals with MEN1 so that diagnosis and familial screening can be readily performed.

## Materials and methods

### Subjects and clinical diagnosis

From a preliminary review of approximately 500 medical records of patients with pituitary neuroendocrine tumor (PitNET) from the database of the Neuroendocrinology Outpatient Clinic of the University Hospital of Brasília, a total of 135 patients met the criteria of at least two affected family members. From this cohort, we have identified 34 families: only four with a phenotype of MEN1 and the other 30 families with the phenotype of familial isolated pituitary adenoma (FIPA). Eleven patients with a clinical diagnosis of MEN1 from these four families were selected.

Clinical diagnosis of MEN1 was defined by the presence of at least two of the three classical tumors (parathyroid, GEP tract, or PitNET). Laboratory tests (calcium, parathyroid hormone (PTH), prolactin, insulin-like growth factor 1 (IGF-1), luteinizing hormone (LH), follicle-stimulating hormone (FSH), estradiol, testosterone, and cortisol), imaging studies, pathological assessment, and relevant clinical events were collected and analyzed through a review of medical records.

The study was carried out in accordance with the Declaration of Helsinki and approved by the Ethics Committee for Research on Human Beings of the Faculty of Health Sciences, University of Brasília. The patients provided their written informed consent to participate in this study.

### MEN1 gene mutation analysis

DNA was extracted from 200 μl of EDTA-whole blood from subjects in an EDTA tube using QIAamp DNA Mini Kit (Qiagen, Hilden, Germany) according to the manufacturer’s instructions and quantified on the Qubit 2.0 fluorometer system using Qubit™ 1X dsDNA HS (Life Technologies, Carlsbad, CA, USA). DNA samples were subjected to a paired-end sequencing process using the NextSeq 500 sequencer (Illumina, San Diego, CA, USA).

Each DNA library was prepared using the KAPA Hyperplus Library Preparation (Roche, Basel, Switzerland), and the coding region of approximately 4,000 genes associated with hereditary diseases and their respective splicing sites were enriched using SeqCap EZ inherited diseases panel (Roche, Basel, Switzerland) and Kapa HyperPrep kit (Roche, Basel, Switzerland). The size and quality of the pre-hybridization and post-hybridization DNA libraries were checked on D1000 ScreenTape using the 4200 TapeStation instrument (Agilent Technologies, Santa Clara, CA, USA) and quantified on the Qubit 2.0 Fluorometer System (Life Technologies, Carlsbad, CA, USA).

The enriched DNA library pool was subjected to paired-end sequencing using the 2 × 75 cycle NextSeq 500/550 V2 midi output kit (Illumina, San Diego, USA) on NextSeq 500 sequencer (Illumina, San Diego, USA).

After demultiplexing using bcl2fastq2 v2.20 conversion software (Base Space, Illumina, San Diego, USA), fastQ files were submitted to DRAGEN Germline 3.5.7 for mapping against the human genome (hg19) and variants calling. Dragen VCF files were uploaded to Varstation (www.varstation.com) for annotation and classification of variants. Twenty-two genes associated with endocrine tumors (*AIP*, *APC*, *CDC73*, *CDKN1B*, *DICER1*, *FH*, *MAX*, *MEN1*, *MET*, *NF1*, *PRKAR1A*, *PTEN*, *RET*, *SDHA*, *SDHAF2*, *SDHB*, *SDHC*, *SDHD*, *TMEM127*, *TP53*, *VHL*, and *WRN*) were filtered for analysis. Variants were classified according to the guidelines of the American College of Medical Genetics and Genomics (ACMG) [PMID 25741868].

## Results

### Clinical features

**Figure 1:**
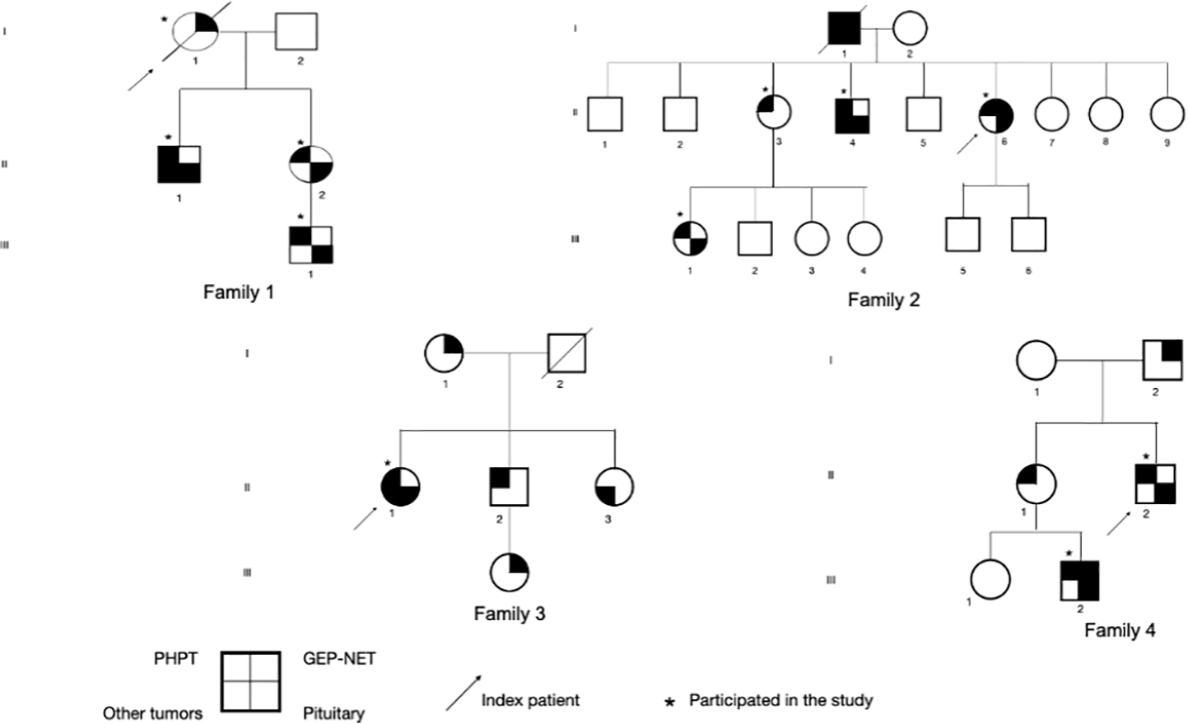
Representation of four pedigrees with clinical/familial MEN1. PHPT, primary hyperparathyroidism; GEP-NET, neuroendocrine tumors of gastroenteropancreatic tract; MEN1, multiple endocrine neoplasia type 1.

We described four families with at least three patients presenting the clinical phenotype of MEN1 (**Figure 1**). The age of the first presentation ranged from 29 to 55 years with a median age of 42 years, and there was no difference in prevalence between genders. All patients that participated in the study (100%) had primary hyperparathyroidism (PHPT), and only three underwent parathyroidectomy (**Table 1**). Five of the six individuals with asymptomatic PHPT who underwent densitometry had low bone mass at diagnosis, and three (individuals III.1 from families 1 and 2 and individual III.2 from family 4) were younger than 30 years.

**Table 1:** Clinical characteristics of subjects from four families with clinical MEN1.

Individual	Sex	Age^a^	PHPT	PIT	Prolactin (ng/ml) IGF-1 (ng/ml)	Tumor dimensions (mm)^b^	GEP-NET	Others
Family 1								
I.1	F	45	+	–	65, 0	–	Metastatic Ga	–
II.1	M	24	+	PRL	1.634, 0	38	–	WDL
II.2	F	28	+	PRL	308, 0	9, 8	–	–
III.1	M	19	+	NFPA	16, 8	9, 7	–	–
Family 2								
II.3	F	49	+	–	10, 0	–	–	–
II.4	M	46	+*	NFPA	12, 0	4, 0	–	Adrenal
II.6	F	43	+	GH/PRL	947, 0	24, 0	Ins	–
III.1	F	26	+	PRL	1.493, 0	23, 0	–	–
Family 3								
II.1	F	38	+*	PRL	453, 0	20, 0	–	Adrenal
Family 4								
II.2	M	55	+*	NFPA	10, 47	41, 0	–	–
III.2	M	29	+	PRL/GH/ACTH	23.730, 0 397, 0**	64, 0	Ga	–

F, female; M, male; PRL, prolactin-secreting; NFPA, non-functioning pituitary adenoma; GH, growth hormone-secreting; ACTH, adrenocorticotropic hormone-secreting; Ga, gastrinoma; Ins, insulinoma; WDL, well-differentiated liposarcoma; MEN1, multiple endocrine neoplasia type 1; PHPT, primary hyperparathyroidism; GEP-NET, neuroendocrine tumors of gastroenteropancreatic tract; PIT, pituitary adenoma.

a At diagnosis (years).

b Largest dimension.

* Underwent partial parathyroidectomy.

** Higher than the normal reference range for age. + (present); - (absent).

**Figure 2:**
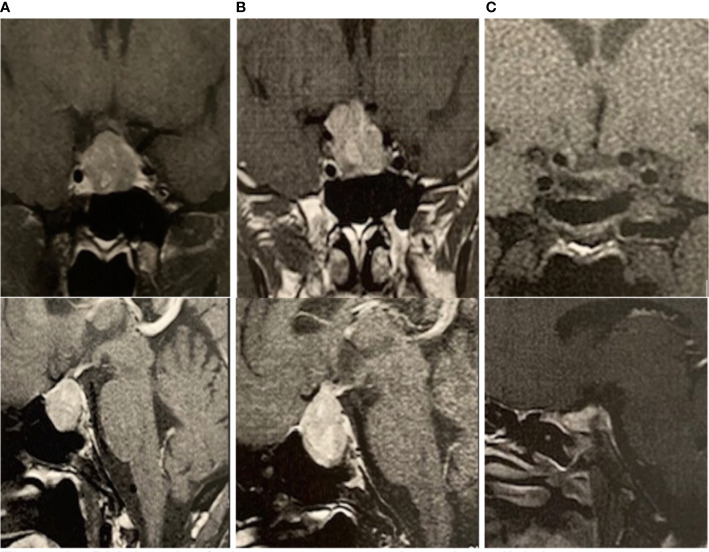
Coronal and sagittal MRI of individual II.1 (family 1). T1-weighted image with a 24 × 19 × 18 mm pituitary macroadenoma **(A)** that evolved after 6 months with hemorrhagic degeneration and compression of the optic chiasm **(B)**. After surgery, a small remnant can be seen in the left half of the anterior pituitary gland **(C)**.

The second most frequent manifestation was a PitNET in nine of the 11 patients (81.8%), with the most common being macroadenomas, which were present in seven individuals (77.8%). Regarding subtypes, prolactinomas were the most common, present in four individuals (44.4%), followed by clinically non-functioning adenomas (33.3%). Three individuals had macroprolactinomas that grew during follow-up, despite a 3.5 mg weekly dose of cabergoline, thus requiring surgical treatment. Patient II.2 (family 1) presented with headache and impaired visual field, with a diagnosis of pituitary apoplexy, and underwent transsphenoidal surgery (**Figure 2**).

**Figure 3:**
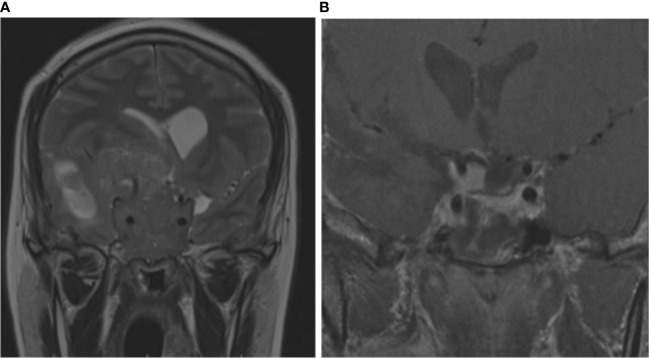
Coronal and sagittal MRI of individual III.2 (family 4). Giant pituitary adenoma with suprasellar extension reaching the right frontoparietal region, with invasion of cavernous sinus and third ventricle **(A)**. Six months after surgery and cabergoline, the tumor exhibited significant shrinkage and necrosis **(B)**.

**Figure 4:**
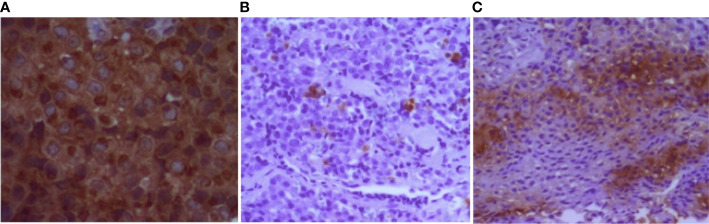
Immunohistochemistry of pituitary tumor (individual III.2, family 4). **(A)** Diffuse positive staining for prolactin. **(B, C)** Strong and focal positive staining for GH and ACTH, respectively. GH, growth hormone; ACTH, adrenocorticotropic hormone.

Individual III.2 (family 4) had a giant pituitary adenoma resulting in intracranial hypertension and underwent craniotomy. He had hyperprolactinemia and IGF-1 higher than the normal reference range for age (**Table 1**). Cabergoline was initiated after surgery at a weekly dose of 3.5 mg. Both prolactin and IGF-1 levels dropped to the normal range after 6 months. Magnetic resonance imaging (MRI) showed a notable reduction in tumor size 6 months after surgery (**Figure 3**). Two individuals had co-secreting adenomas with one having immunohistochemistry showing positive staining for prolactin, growth hormone (GH), and adrenocorticotropic hormone (ACTH) (**Figure 4**).

Only three patients had GEP tract tumors—two gastrinomas and one insulinoma—who underwent surgical resection. The only patient in our study who died was the index case (I.1) of family 1 who was diagnosed with metastatic gastrinoma. In this family, an uncommon association with well-differentiated liposarcoma was found in patient II.2. Two individuals had adrenal tumors with dimensions greater than 4 cm. Individual II.4 (family 2) had a bilateral lesion and subclinical hypercortisolism, and individual II.1 (family 3) had a non-functioning unilateral lesion (**Figure 1**), both treated surgically.

### Genotyping analysis

**Figure 5:**
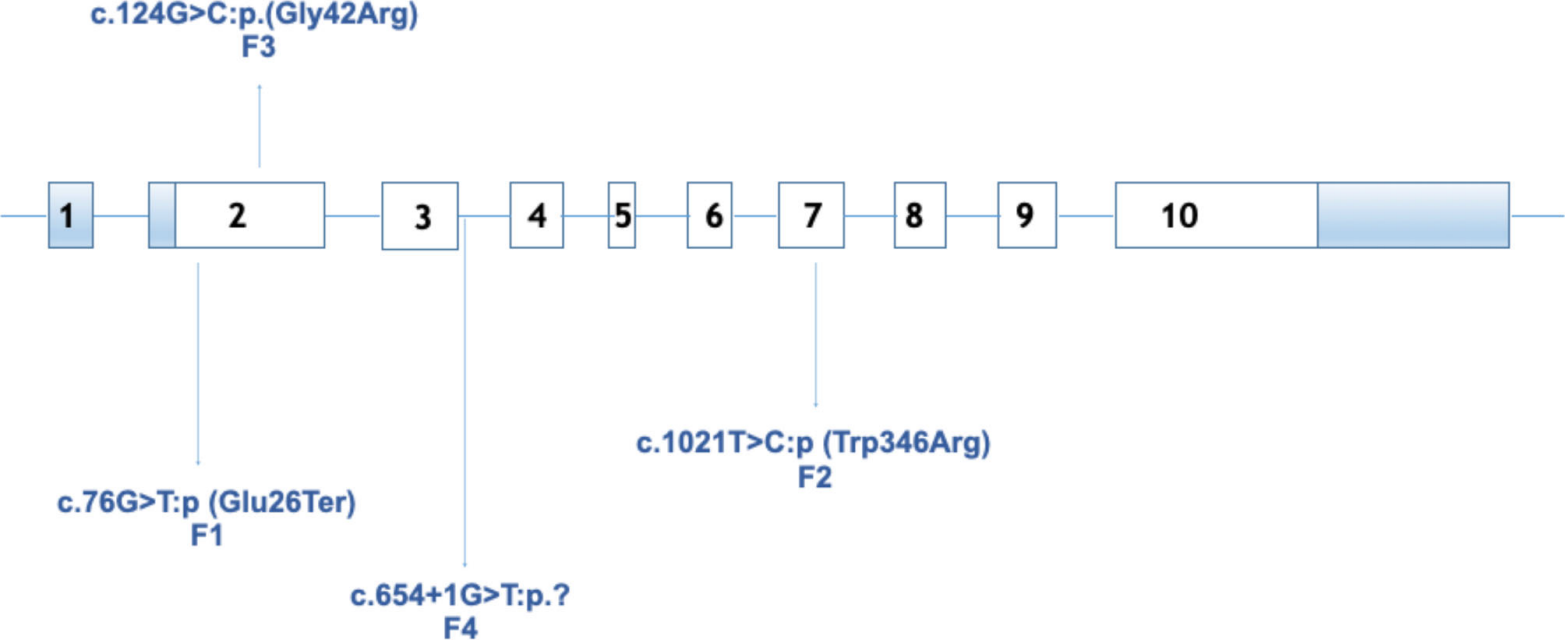
Distribution of germline mutations in *MEN1* gene identified in individuals from each family of our study (NM_130799.3). F1, family 1; F2, family 2; F3, family 3; F4, family 4.

Variants in *MEN1* gene were identified in all families. In individual II.1 (family 1), a non-sense mutation (NM_130799.3:c.76G>T:p.Glu26Ter) has been found in exon 2. Missense mutations have been detected in individuals II.6 (family 2) and II.1 (family 3) (NM_130799.3:c.1021:p.Trp341Arg in exon 7 and NM_130799.3:c.124G>C:p.Gly42Arg in exon 2, respectively). Another variant has been identified in the splicing site (NM_130799.3:c.654+1G>T) in individual II.2 (family 4) (**Figure 5**). Allelic variants found in individuals II.1 (family 1), II.6 (family 2), and II.2 (family 4) were considered pathogenic after applying ACMG variant classification rules (12). A novel variant was found in individual II.1 (family 3) and classified as probably pathogenic (**Table 2**).

**Table 2:** Mutation analysis of each family with MEN1 (NM_130799.3).

Patient	Sex/age^a^	Exon/Intron	Variant	Effect	First description	ACMG rules	ACMG classification
II.1 (F1)	M/24	2	c.76G>T p.Glu26Ter	Non-sense	(9)	PVS1 PS4 PM2 PP3	P
II.6 (F2)	F/43	7	c.1021T>C: p.Trp341Arg	Missense	(10)	PS1 PS4 PM2 PM1 PP2 PP3	P
II.1 (F3)	F/38	2	c.124G>C p.Gly42Arg	Missense	This study	PM1 PM2 PM5 PP2 PP3	LP
II.2 (F4)	M/55	4	c.654+1G>T	Splicing	(11)	PVS1 PS4 PM2 PP2 PP5	P

F, female; M, male; P, pathogenic; LP, likely pathogenic; F1, family 1; F2, family 2; F3, family 3; F4, family 4; ACMG, American College of Medical Genetics and Genomics; MEN1, multiple endocrine neoplasia type 1.

a At diagnosis (years).

## Discussion

This study was the first to perform both clinical and molecular descriptions of four families with MEN1 in a follow-up at a tertiary center in Brasília. Patients with MEN1 have a reduced life expectancy when compared to individuals with sporadic tumors (1). The prognosis improves considerably when these tumors are identified at an early stage, especially in patients who are asymptomatic (2). In this regard, it is necessary for an interdisciplinary team that can clinically recognize the phenotypic variability found in these individuals, as well as perform family genetic screening.

The median age at diagnosis of the index cases was 45 years, which suggests a certain delay in diagnosis. It has also been observed in other countries and can occur because of both a delay in identifying the index case and the lag time to complete the family screening (13). A study using the database of patients with MEN1 in Japan showed that the mean age at diagnosis occurred in the fourth decade in 50% of cases (14). Another study using an Italian database also found that the mean age at diagnosis was also around the fourth decade (15).

Studies in patients with MEN1 have already shown that women aged between 20 and 35 years have a higher incidence of osteopenia and osteoporosis when compared to the general population of the same age group (16–18). PHPT was present in all patients of our study, and three asymptomatic individuals under 30 years old had low bone mass at diagnosis. A Brazilian study demonstrated impairment of bone mass in half of the individuals under the age of 30 years, despite being more frequent and severe in patients over 50 years old (19). Early development of PHPT, occurring about three decades before sporadic cases, leads to prolonged and persistent exposure to high levels of PTH. Therefore, peak bone mass is compromised, and, consequently, the risk of fractures increases (20).

Although PHPT is the first clinical manifestation in almost 90% of cases (15, 21), PitNETs were the first and most common manifestation in our series, occurring in five out of 11 individuals (45.5%). We believe that this was due to the fact that patients were referred to our neuroendocrinology outpatient clinic. Most of them had macroadenomas (77.8%), which are more common in individuals with MEN1 and generally present morphological invasion of adjacent structures compared to sporadic cases (22–24).

Prolactinomas were the most common, present in four individuals (44.4%), followed by non-functioning pituitary adenoma in three individuals (33.3%). The literature shows that prolactinomas are the most prevalent in the context of MEN1 (~65%), followed by somatotropinoma, ACTH-secreting adenoma, and non-functioning adenomas (21, 25). Some studies have shown that plurihormonal adenomas are more frequent in the context of MEN1 when compared to sporadic pituitary adenomas. Unusual associations may occur, such as the secretion of prolactin and ACTH (26, 27), which was present in one of our patients who had immunohistochemistry showing positive staining for prolactin, GH, and ACTH (individual III.2 of family 4).

A higher frequency of GEP tract tumors is expected in these individuals since it is the second most common manifestation in MEN1 (1, 3). However, only three individuals had GEP tract tumors: two gastrinomas and one insulinoma. It is possible that imaging studies missed some of these tumors, especially non-functioning ones. Furthermore, endoscopic ultrasound was not available in all radiology services.

Adrenal lesions have been described in approximately 36% to 73% of patients with MEN1 (28, 29). They are usually diagnosed as incidentalomas during radiological screening, and most are non-functioning (30). Previous studies have shown that most of these lesions are less than 4 cm in diameter (29, 31, 32). Two individuals had adrenocortical adenomas with dimensions greater than 4 cm (II.4 of family 2 and II.1 of family 3). Probably, the patients in our study had a later diagnosis, which may justify this finding.

A genotype–phenotype correlation in MEN1 has been difficult to demonstrate even among family relatives harboring the same variants (33). It appears that mutations play an uncertain role in the clinical features of the disease, such as the age of onset, recurrence, or aggressiveness markers (34, 35). By observing the clinical manifestations of the 11 participants in our study, we noticed the heterogeneity of phenotypes presented among members of the same family. Nevertheless, genetic evaluation reduces the morbidity and mortality of individuals such as MEN1, as it allows to identify cases in the clinical spectrum and to receive treatment in the early stages of the disease (4, 6).

There are still little data about the clinical and genetic features of MEN1 in Brazil. Therefore, most patients may be symptomatic at diagnosis. Some studies report clinical and genetic screenings of patients with MEN1 conducted at Hospital das Clínicas in São Paulo, which has become one of the reference services for this disease in Brazil (8, 36, 37). In our center, as a University Hospital, we offer genetic testing to all patients with clinical evidence of FIPA, MEN1, and MEN2.

Some studies used the Sanger method or multiplex binding-dependent probe amplification (MLPA) for *MEN1* gene screening (38–40). We chose to perform targeted HTS to identify the variants because it analyses multiple coding regions and splicing sites simultaneously of *MEN1* gene and other genes associated with MEN1-like phenotypes as *CDKN1B* and *AIP*, respectively, causing MEN4 and FIPA. Therefore, information acquisition occurs with greater speed and lower estimated cost when compared to the Sanger method (7, 8).

A non-sense variant has been identified in family 1 (c.76G>T:p.Glu26Ter), causing an early stop codon in transcription and thus reducing the reading frame by more than 90%. There is a loss of functional domains, such as nuclear localization sequences (NLSs) located in the C-terminal portion of MENIN, NLS-1 (codons 479–497), and NLS-2 (codons 588–608). In addition, there is a loss of interaction sites of MENIN and JunD protein, a member of the AP1 transcription factor family (protein 1 activator) (41). This variant was first described in a Danish family with PHPT and carcinoid tumor of the duodenum (9). There is also a description of the same mutation in a Hungarian family with two individuals who had PHPT and prolactinoma (42), a similar phenotype found in two individuals (II.1 and II.2) of family 1 in our study. The novel missense variant in exon 2 (c.124G>C:p.Gly42Arg) identified in family 3 seems to also affect the binding site of MENIN protein with JunD. There are descriptions of mutations that occurred at the same position but with different amino acids (43, 44).

Angiofibromas, collagenomas, and lipomas are common in MEN1 (1, 45). The incidence of lipomas can range from 0.9% to 34% in these patients (46, 47) and may present with an atypical location such as in the intrathoracic region (48). Well-differentiated liposarcomas are malignant mesenchymal neoplasms that usually affect proximal regions of the limbs and the retroperitoneum, occurring more frequently between the fifth and seventh decades of life (49). In addition to lipomas in the right thoracic and clavicular regions, patient II.1 of family 1 had well-differentiated liposarcoma in the right shoulder, an association that has been previously reported in a single case (50). Amplifications involving the region of chromosome 12 (12q13-15) have been found in these tumors by some authors, mainly involving the oncogenes MDM2 and CDK4 (51).

MENIN also interacts with Smad3, one of the receptor-regulated elements that mediate TGF-b (52). It seems that impairment of the MENIN protein blocks the transcriptional effects mediated by Smad3 and TGF-b, which can lead to inadequate cell growth and tumor onset (5, 52). The variant found in family 4 (c.654+1G>T) occurs at a splicing site leading to an early stop codon, and it has been described in several individuals with MEN1. The first report was in an Australian family (11) and later described in a Brazilian study in which the patient had PHPT, prolactinoma, and gastrinoma (53), a similar phenotype to that found in one of the individuals in family 4 (III.2). A case report recently published also describes a young individual with the same variant in *MEN1* who had a giant pituitary adenoma and intracranial hypertension (54), similar to individual III.2 (family 4). Functional studies using lymphoblastoid cell lines from an individual with PHPT and gastrinoma were performed to assess the pathophysiological implications of this variant. It has been shown that the mutant MENIN was not able to bind to Smad3, resulting in a blockage in the TGF-b signaling pathway and, consequently, affecting its inhibitory actions on cell growth (55).

A missense variant in exon 7 has been identified in family 2 (c.1021:p.Trp341Arg), which was previously described in two individuals with clinical MEN1: one with PHPT, GEP tract tumor and a non-functioning adrenal tumor (10) and another with PHPT, GEP tract tumor, and pituitary tumors (56). There is also another report of the same mutation in one individual with familial isolated hyperparathyroidism (FIHP) (34). Although FIHP is described as a distinct genetic entity, it is also believed to be a variant of other familial neoplastic syndromes in which PHPT is the main clinical manifestation, as in MEN1 (57). This milder MEN1 phenotype characterized by FIHP has been described in some families and may be more related to mutations located between exons 3 and 7 of the MENIN protein gene (58, 59). Even though all individuals in family 2 who participated in the study had PHPT, they also had other endocrine tumors that are part of the clinical condition of MEN1, such as pituitary adenomas secreting prolactin and GH, insulinoma, and adrenal tumors.

Our study has limitations, as we evaluated a small number of families. Also, there was heterogeneity in complementary exams performed during screening, as many were carried out in different radiology and pathology services. Although uncommon, we did not investigate deletions or duplications since multiplex ligation-dependent probe amplification (MLPA) or copy number variation (CNV) analysis was not performed. However, the study allowed the genotypic and phenotypic characterization of families with MEN1 in a follow-up at a tertiary center in Brasília.

In conclusion, MEN1 is a rare condition expressed by variable combinations of endocrine and non-endocrine tumors. Due to its heterogeneous phenotype, the diagnosis is late in most cases. Hence, these patients may already present with complications resulting from these tumors, which increases morbidity and mortality. Multicenter and prospective studies are important for a better understanding of the clinical and molecular characteristics of patients from our region to improve care in terms of diagnosis, treatment, and follow-up.

## Data availability statement

The data presented in the study are deposited in the BioSample database repository, using the accession number PRJNA923744; The links to the individual samples are as follows: https://www.ncbi.nlm.nih.gov/sra/SRX19034472; https://www.ncbi.nlm.nih.gov/sra/SRX19034471; https://www.ncbi.nlm.nih.gov/sra/SRX19034470; https://www.ncbi.nlm.nih.gov/sra/SRX19034469.

## Ethics statement

The studies involving human participants were reviewed and approved by Ethics Committee for Research on Human Beings of the Faculty of Health Sciences, University of Brasília. The patients/participants provided their written informed consent to participate in this study.

## Author contributions

IM and LN were major contributions in writing the manuscript. IM, LN, LV, LdS and LdC analyzed the patient data. GB, PM and TR performed and analyzed the molecular data. All authors contributed to manuscript revision, read, and approved the submitted version.
